# A head-to-head comparison of myocardial strain by fast-strain encoding and feature tracking imaging in acute myocardial infarction

**DOI:** 10.3389/fcvm.2022.949440

**Published:** 2022-07-28

**Authors:** Walid El-Saadi, Jan Edvin Engvall, Joakim Alfredsson, Jan-Erik Karlsson, Marcelo Martins, Sofia Sederholm, Shaikh Faisal Zaman, Tino Ebbers, Johan Kihlberg

**Affiliations:** ^1^Department of Internal Medicine, Ryhov County Hospital, Region Jönköping County, Jönköping, Sweden; ^2^Department of Health, Medicine and Caring Sciences, Linköping University, Linköping, Sweden; ^3^Department of Clinical Physiology in Linköping and Department of Health, Medicine and Caring Sciences, Linköping University, Linköping, Sweden; ^4^Center for Medical Imaging Science and Visualization, Linköping University, Linköping, Sweden; ^5^Department of Cardiology in Linköping and Department of Health Medicine and Caring Sciences, Linköping University, Linköping, Sweden; ^6^Department of Radiology in Linköping and Department of Health Medicine and Caring Sciences, Linköping University, Linköping, Sweden

**Keywords:** cine magnetic resonance imaging, myocardial ischemia, ST elevation myocardial infarction, myocardial stunning, left ventricular dysfunction, left ventricular remodeling

## Abstract

**Background:**

Myocardial infarction (MI) is a major cause of heart failure. Left ventricular adverse remodeling is common post-MI. Several studies have demonstrated a correlation between reduced myocardial strain and the development of adverse remodeling. Cardiac magnetic resonance (CMR) with fast-strain encoding (fast-SENC) or feature tracking (FT) enables rapid assessment of myocardial deformation. The aim of this study was to establish a head-to-head comparison of fast-SENC and FT in post-ST-elevated myocardial infarction (STEMI) patients, with clinical 2D speckle tracking echocardiography (2DEcho) as a reference.

**Methods:**

Thirty patients treated with primary percutaneous coronary intervention for STEMI were investigated. All participants underwent CMR examination with late gadolinium enhancement, cine-loop steady-state free precession, and fast-SENC imaging using a 1.5T scanner as well as a 2DEcho. Global longitudinal strain (GLS), segmental longitudinal strain (SLS), global circumferential strain (GCS), and segmental circumferential strain (SCS) were assessed along with the MI scar extent.

**Results:**

The GCS measurements from fast-SENC and FT were nearly identical: the mean difference was 0.01 (2.5)% (95% CI – 0.92 to 0.95). For GLS, fast-SENC values were higher than FT, with a mean difference of 1.8 (1.4)% (95% CI 1.31–2.35). Tests of significance for GLS did not show any differences between the MR methods and 2DEcho. Average strain in the infarct-related artery (IRA) segments compared to the remote myocardium was significantly lower for the left anterior descending artery and right coronary artery culprits but not for the left circumflex artery culprits. Fast-SENC displayed a higher area under the curve for detecting infarcted segments than FT for both SCS and SLS.

**Conclusion:**

GLS and GCS did not significantly differ between fast-SENC and FT. Both showed acceptable agreement with 2DEcho for longitudinal strain. Segments perfused by the IRA showed significantly reduced strain values compared to the remote myocardium. Fast-SENC presented a higher sensitivity and specificity for detecting infarcted segments than FT.

## Introduction

Coronary artery disease is a major cause of heart failure worldwide, as more patients now survive myocardial infarction (MI) due to improvements in prevention as well as in the availability of primary percutaneous coronary intervention (pPCI) in the case of ST-elevation MI (STEMI) (1–4). Left ventricular (LV) adverse remodeling, which may develop post-MI, is a complex process, initiated by scarring, which results in myocardial functional and anatomical deterioration (1, 2, 4). A myocardial scar is characterized by wall thinning and abnormal wall motion. On a global level, increased LV volumes, partial bulging of the LV wall, and reduced left ventricular ejection fraction (LVEF) are typical characteristics of remodeling (4, 5). Beyond LV volumes and LVEF, measurements of myocardial deformation, frequently denominated “strain,” can add information on the reduction in myocardial performance not yet visible in the gold standard LVEF (6). Two-dimensional echocardiography (2DEcho) studies have demonstrated that strain may predict adverse remodeling (4, 7). 2DEcho is a time and cost-effective standard procedure in post-MI care but is limited by the skills of the operator and problems evaluating segments due to artifacts and pulmonary shadowing (8). Cardiac magnetic resonance (CMR) is considered the reference method for the assessment of LV anatomy and function but has some drawbacks, such as being time-consuming, unsuitable for claustrophobic patients, and often requiring the use of gadolinium contrast, which is contraindicated in renal failure (9–12). Late gadolinium enhancement (LGE) is the method of choice for detecting myocardial necrosis and scarring (2, 9, 13, 14). LGE imaging is commonly performed about 10–20 min after contrast injection to detect injured myocytes, infarct scar area, and its transmural extent, features that are not available with other imaging methods (2, 9, 13). Strain assessment by feature tracking (FT) or fast-strain encoding (fast-SENC) CMR may add to the evaluation of patients with acute MI by identifying individuals who could be at risk of developing adverse remodeling (10, 15, 16). Both techniques can assess strain in the longitudinal and circumferential directions, which has been shown to predict adverse remodeling of the LV (16–19). The techniques used in FT and fast-SENC are discussed in Amzulescu et al. (6). FT is computed on cine-loops which are part of the standard balanced steady-state free precession (bSSFP) CMR examination (8, 16). These segmented 2D cine-loops are acquired over the entire heart cycle, usually “averaged” from 5 to 10 heartbeats, which makes deformation measurement possible for each time step (6, 16, 20). Feature tracking (FT) uses either optical flow technology or non-rigid elastic registration (21). Fast-SENC utilizes parallel tags and needs only a single heartbeat for image acquisition, and post-processing can be completed in <2 min. This may eliminate the need for breath-holding, which is especially valuable in patients with respiratory diseases. In patients with cardiac arrhythmia, a single heartbeat image acquisition will also result in fewer artifacts (22).

The aim of this study was to establish a head-to-head comparison of myocardial strain assessment, in both longitudinal and circumferential directions between fast-SENC and FT in STEMI patients immediately post-pPCI using echocardiographic speckle-tracking strain as the reference.

## Materials and methods

### Study population

**Figure 1:**
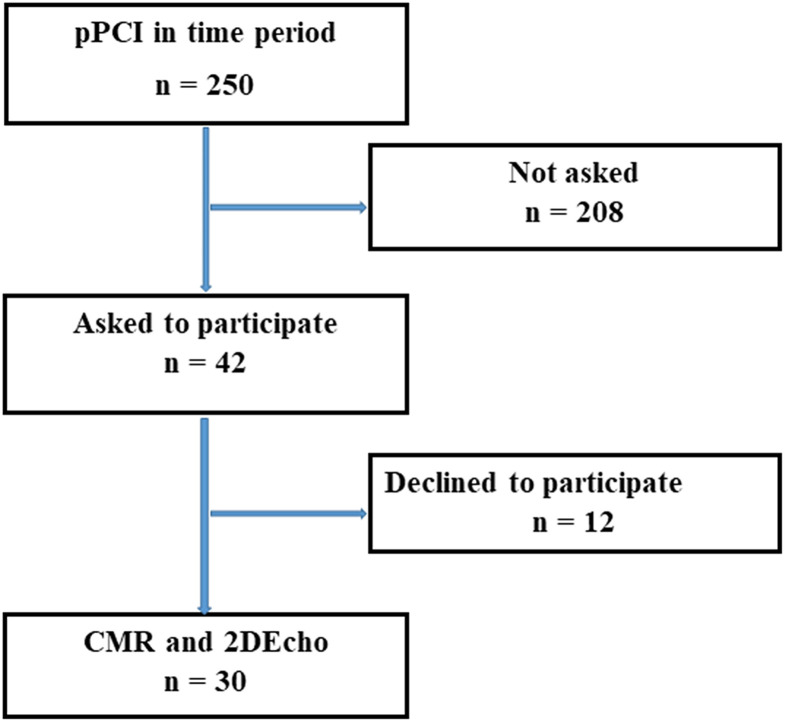
Flowchart of study design.

Patients with STEMI, treated with pPCI were offered CMR and 2DEcho within 2 days, while still in the hospital, between 4 November 2019 and 16 November 2020. In this time span, a total of 250 patients were treated with pPCI for STEMI at our hospital. Forty-two patients were asked to participate, 12 declined, and 30 were finally enrolled in the study after giving written and oral consent, see Figure 1. The study complied with the Declaration of Helsinki and with agreements on Good Clinical Practice. The study protocol was approved by the Swedish Ethical Review Authority in Uppsala, registration number 2019-00480.

### CMR acquisition and post-processing

CMR including cine bSSFP, LGE, and fast-SENC was acquired on a 1.5T scanner (Achieva d-Stream, Philips Healthcare, Best, the Netherlands). The fast-SENC acquisition had a voxel size of 4.0 × 4.0 × 10 mm^3^, which was reconstructed to 1.0 × 1.0 × 10 mm^3^. The acquisition length was one cardiac cycle for each cardiac view, requiring a 1-s breath hold at a heart rate of 60 beats per minute, enabling the images to be reconstructed into 22 phases. The following acquisition parameters were used for fast-SENC: repetition time (TR) = 11 ms, echo time (TE) = 0.7 ms, flip angle = 30°. Fast-SENC strain analysis was performed in the MyoStrain software (Myocardial Solutions Inc. v 5.1.4, Morrisville, NC, USA), the technique utilizes tags parallel to the acquired image and is an adaptation of SENC, enabling the acquisition of cardiac deformation in a single heartbeat. Longitudinal strain (LS) was derived from three different short-axis (SA) views, covering the left ventricle at the basal, midventricular, and apical level. Circumferential strain (CS) was derived from two-, three- and four-chamber long axis (LA) views. The LV 17-segment model of the American Heart Association (AHA) was used, excluding the SA apical segment (23). The time required for performing post-processing was recorded, and the time span of the acquisitions was obtained from the DICOM header of the stored images. For FT, bSSFP images were acquired with a spatial resolution of 1.2 × 1.2 × 8 mm^3^ and reconstructed into 30 cardiac phases. The FT algorithm is based on non-elastic registration of segmented endo- and epicardial surfaces with the deformation field being tracked over time. The following FT acquisition parameters were used: TR = 3.3 ms, TE = 1.6 ms, and flip angle = 60°. The typical breath-hold duration was 9 s for each view, at a heart rate of 60 beats per minute. Three different LA (two-, three-, and four-chamber) and SA (at basal, midventricular, and apical levels) images were obtained, excluding the SA apical segment. CS was derived from the SA segments and LS from the LA image segments according to the AHA model (23). The images were segmented for volume, left ventricular mass (LVM), and MI scar in the Segment software (v 2.2 R7056, Medviso AB, Lund, Sweden), which also included a module that was used for FT strain analysis (non-rigid elastic registration). LGE was acquired in the same views as the cine images, using the PSIR-technique with a resolution of 1.5 × 1.5 × 10 mm^3^ with a typical breath-hold duration of 12 s for each image. All strain values were evaluated at end-systole, which was determined from aortic valve closure. One observer performed segmentation for FT strain, LV volume, and MI scar analysis. A “scar” segment was defined if the LGE-positive area was >1%. The processing time for FT and fast-SENC was recorded for 10 randomly selected patients. For analysis of intraobserver and interobserver reproducibility, patients were re-analyzed twice by one CMR operator and once by another CMR operator, both experienced in the field. Operators were certified for the acquisition and analysis of fast-SENC by the vendor.

## Echocardiography

Standard transthoracic 2DEcho was recorded for clinical routine evaluation by clinically experienced technicians with the patient in the left lateral decubitus position. Speckle tracking 2DEcho allows for the evaluation of myocardial deformation by assessing the movements of small natural acoustic markers during a heart cycle. A Vivid E-95 Ultrasound System (GE Vingmed Ultrasound; Horten, Norway) equipped with a 4Vc-probe was used for assessment of myocardial function and structure via the parasternal long axis, the apical two-, three-, and four-chamber views and when necessary also the subcostal views. End-systolic global longitudinal strain (GLS) was analyzed offline using the 2DS tool in EchoPAC PC Integrated version 203.74 (GE Ultrasound, Horten, Norway), by an echocardiographic specialist experienced in speckle tracking.

## Comparison methodology

Global circumferential strain (GCS) and global longitudinal strain (GLS) derived from FT and fast-SENC were compared head-to-head. Speckle tracking end-systolic GLS from the 2DEcho gray scale was calculated for reference. All strains were correlated to LVEF_CMR_, MI scar, and its segmental extent (“transmurality”). The diagnostic performance of segmental circumferential strain (SCS) and segmental longitudinal strain (SLS) was based on individual segments and the regional strain was calculated by assigning myocardial segments to the three major coronary artery perfusion territories according to Cerqueira et al. (23). Strain in segments belonging to the infarct-related artery (IRA) was compared to remote myocardial segments. The detection of scar segments based on strain results was presented as the area under the curve (AUC) from receiver operating characteristics curve (ROC) analysis. Sensitivity was calculated at a specificity of 80% for the detection of any infarcted segment as well as for segments with transmurality >50%.

## Statistical analysis

Analysis was performed using SPSS 27 (IBM Inc, Armonk, New York, USA). Continuous variables were presented as mean with SD (in parenthesis). Differences in continuous variables were tested with the analysis of variance non-parametric Friedman's Chi square test, where the level of significance was set to *p* < 0.01. Pearson correlation coefficients (*ρ*, df) where df = *N*−2, were calculated to express the degree of linear association between the variables. The correlation hypothesis tested was that *ρ* = 0 vs. *ρ* ≠ 0 with a significance level set to *p* < 0.01. The intraclass correlation coefficient (ICC) was calculated, scatterplot graphs were drawn to depict the linear relationship between the variables and boxplots were created to illustrate the distribution of myocardial strain. Bland–Altman difference plots were presented to evaluate the agreement between the CMR methods.

## Results

### Scar and ejection fraction

The subjects were enrolled and treated with pPCI after identification of the culprit artery in each case. The cohort displayed a median door-to-balloon time of 67 min. Average scar size was 15 (9) % of LVM with a median Troponin-T of 1,640 ng/l, equivalent to 164 × upper level of normal. LGE revealed scar in 240 out of 510 segments (47%) with 122 segments having scar transmurality < 25%, 78 segments between 25 and 49%, and only 40 segments had a transmurality ≥ 50%. In 13 patients the LVEF_CMR_ was little affected, LVEF_CMR_ ≥ 50%. Patients with maintained LVEF_CMR_ had smaller scar size 10 (5) % than those with depressed LVEF_CMR_ < 50% whose scar size was 19 (10) %, (*p* < 0.01). Patient demographics and CMR imaging characteristics are presented in Table 1.

**Table 1:** Patient characteristic.

**Patient demographics (*****n*** = **30)**	**Mean (** ***SD*** **)**
Men/women	22/8
Age (years)	69 (10)
Height (cm)	173 (11)
Weight (kg)	81 (16)
BMI (kg/m^2^)	27 (5)
eGFR (ml/min)	79 (24)
**Cardiovascular risk profile**	
Family history of cardiovascular disease	4
Diabetes	9
Hyperlipidemia	17
Hypertension	21
History of MI	10
Previously treated PCI	8
**Culprit artery**	
LAD	13
LCX	6
RCA	11
**Cardiac magnetic resonance imaging characteristics**	
**Left ventricular morphology**	
LVEDV (ml)	159 (43)
LVESV (ml)	84 (36)
LVSV (ml)	75 (19)
LV Mass (g)	124 (27)
LVEF %	48 (9)
MI scar %	15 (9)

Means with standard deviation (SD) in parentheses. For abbreviations, please see text.

## Myocardial strain

**Figure 2:**
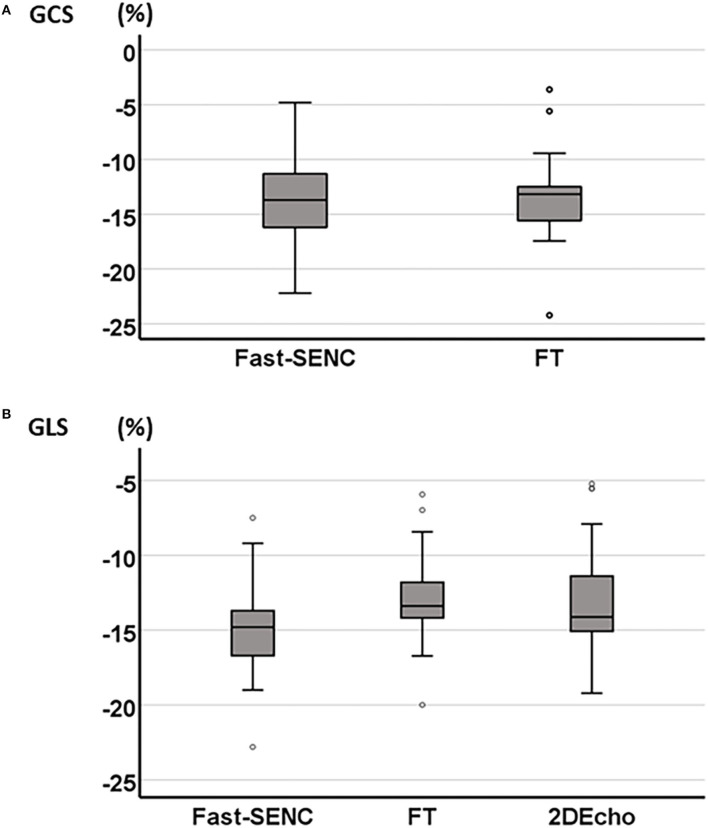
Boxplot of mean GCS derived from fast-SENC and FT **(A)** and mean GLS derived from fast-SENC, FT, and 2DEcho **(B)**.

**Figure 3:**
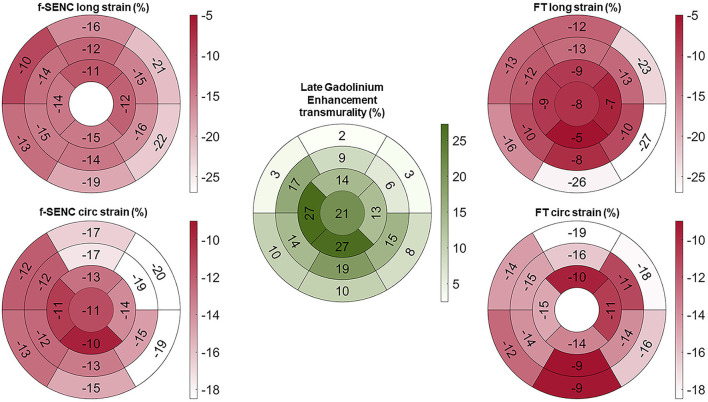
Bull's eye of mean longitudinal strain (long) and global circumferential strain (circ) of fast-SENC and FT with myocardial injury detected with late gadolinium enhancement (LGE).

**Figure 4:**
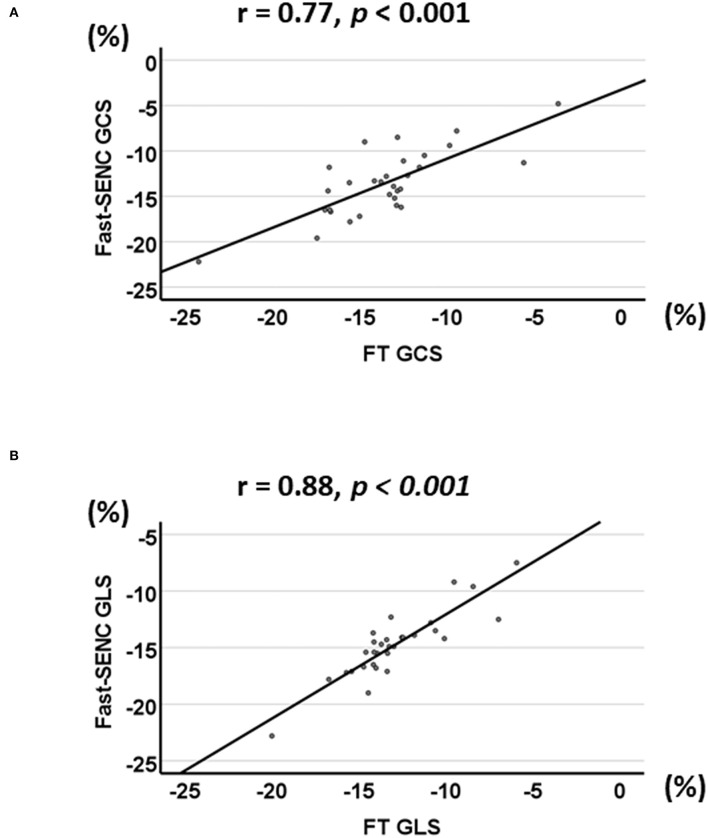
Linear correlation (*r*) between fast-SENC and FT for GCS **(A)** and GLS **(B)**.

**Figure 5:**
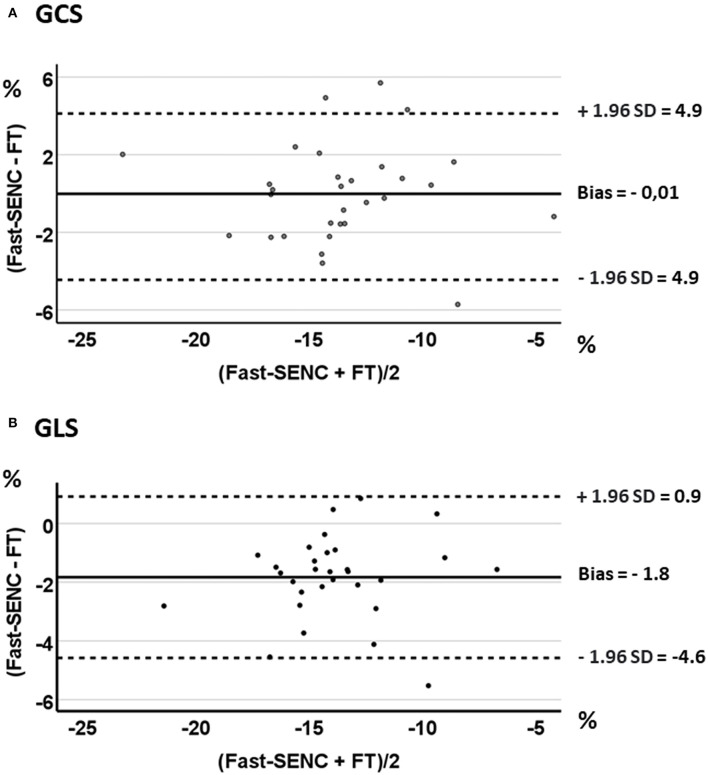
Bland-Altman plots with limits of agreement (1.96 SD) for fast-SENC and FT for GCS **(A)** and GLS **(B)**.

**Figure 6:**
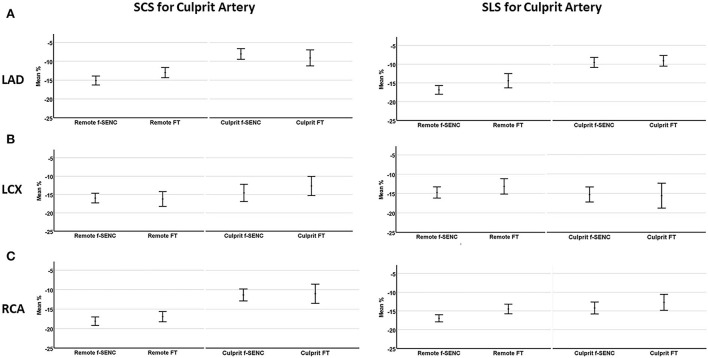
Mean strain of culprit artery segments and remote myocardium for left anterior descending artery **(A)**, left circumflex artery **(B)**, and right coronary artery **(C)**. Segmental circumferential strain (SCS) and segmental longitudinal strain (SLS) are presented for each infarcted related artery.

**Figure 7:**
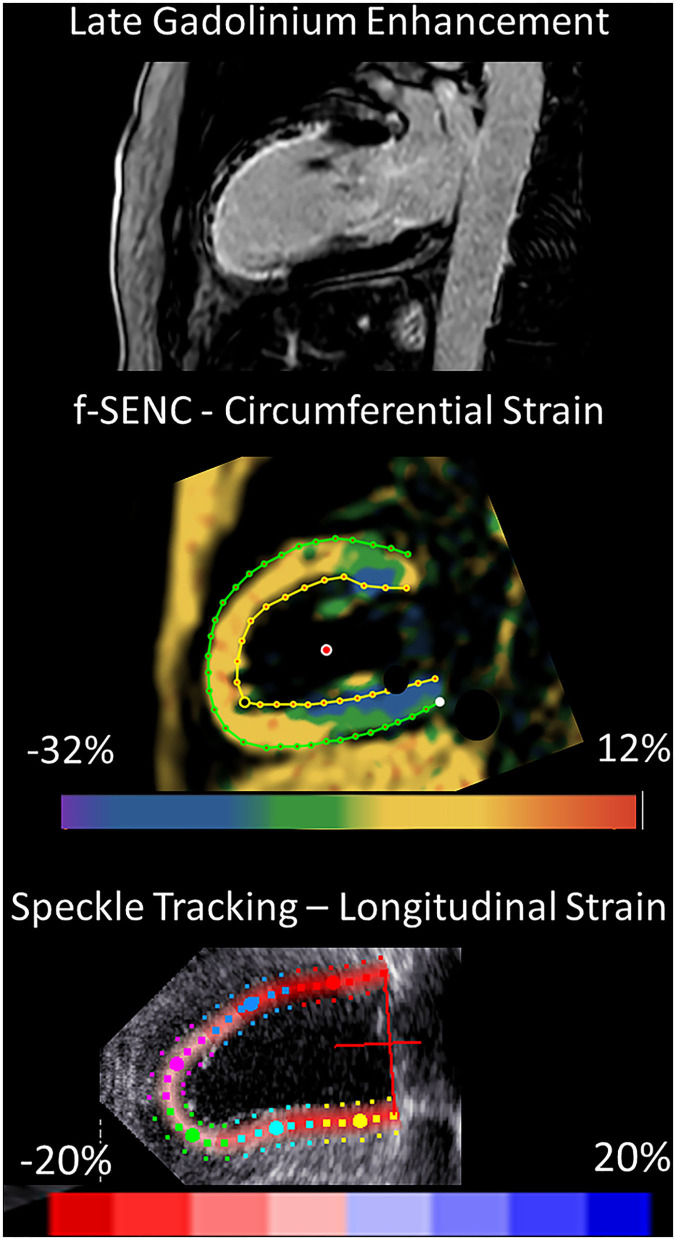
2-chamber view of an extensive anterior infarction with the transmural extent of late gadolinium enhancement and zones of no-reflow in the superior viewport, distinctly positive circumferential strain by fast-SENC in the scar area (yellow, middle viewport) and the corresponding speckle tracking longitudinal strain from 2DEcho (pale pink, lower viewport).

Strain comparisons are given in terms of “higher” when more negative, and “lower” when less negative, according to Voigt et al. (24). The GCS measurements from fast-SENC and FT were nearly identical, with a mean difference of 0.01 (2.5)% (95% CI−0.92 to 0.95). For GLS, fast-SENC values were higher than FT with a mean difference of 1.8 (1.4)% (95% CI 1.31–2.35), Table 2 and Figures 2, 3. Statistical testing for GLS did not show significant differences between fast-SENC or FT and 2DEcho (*p* > 0.01). The correlations between GCS or GLS from the two myocardial deformation techniques and MI scar, LVEDV_CMR_ and LVEF_CMR_ are shown together with Bland–Altman graphs in Table 3 and partly in Figures 4, 5. Average strain in the IRA segments compared to the remote myocardium was significantly (*p* < 0.001) lower for left anterior descending artery (LAD) and right coronary artery culprits but not for left circumflex artery culprits, Table 4 and Figure 6. The average SCS from fast-SENC showed a higher correlation to MI scar than the average SCS for FT for each IRA segment distribution (*p* < 0.001). The highest correlation factor was computed for the average SCS and scar in the LAD region (*ρ* = 0.65, *p* < 0.001). In general, correlations were higher for fast-SENC in both strain directions compared to FT and 2DEcho, except for SLS vs. scar in LAD segments, Table 5. Figure 7 shows a two-chamber view example of an extensive anterior infarction with LGE, fast-SENC_CMR_, and speckle tracking strain from 2DEcho.

**Table 2:** Global circumferential and longitudinal strain.

**Myocardial strain direction**	**Mean % (SD)**	**95% CI for mean**
**GCS**		
Fast-SENC	−13.6 (3.7)	−14.9 to −12.2
FT	−13.6 (3.7)	−15.0 to −12.2
**GLS**		
Fast-SENC	−14.8 (2.9)	−15.9 to −13.7
FT	−13.0 (2.8)	−14.0 to −11.9
2DEcho	−13.3 (3.7)	−14.7 to −11.9

Global circumferential strain (GCS) and global longitudinal strain (GLS) derived from fast-SENC, FT and 2DEcho. Means and standard deviation (SD) in parenthesis with 95% confidence intervals are presented.

**Table 3:** Correlation chart of global strain.

**Correlation variables**	***r*** **(df)**	***p***
**GCS**		
Fast-SENC vs. FT	0.77 (28)	<0.01
**GLS**		
Fast-SENC vs. FT	0.88 (28)	<0.01
2DEcho vs. fast-SENC	0.65 (27)	<0.01
2DEcho vs. FT	0.75 (27)	<0.01
**GCS vs. MI scar**		
Fast-SENC	0.65 (28)	<0.01
FT	0.54 (28)	<0.01
**GLS vs. MI scar**		
Fast-SENC	0.41 (28)	0.02
FT	0.47 (28)	<0.01
2DEcho	0.53 (27)	<0.01
**GCS vs. LVEF**		
Fast-SENC	−0.32 (28)	0.09
FT	−0.22 (28)	0.25
**GLS vs. LVEF**		
Fast-SENC	−0.19 (28)	0.31
FTd	−0.30 (28)	0.11
2DEcho	−0.35 (27)	0.06
**GCS vs. LVEDV**		
Fast-SENC	0.33 (28)	0.08
FT	0.31 (28)	0.09
**GLS vs. LVEDV**		
Fast-SENC	0.09 (28)	0.63
FT	0.18 (28)	0.34
2DEcho	0.09 (27)	0.64

Pearson correlation coefficients (r) with degrees of freedom (df = n-2), for global circumferential strain (GCS) and global longitudinal strain (GLS) correlated to myocardial infarction (MI) scar, left ventricular ejection fraction (LVEF), and left ventricular end-diastolic volume (LVEDV).

**Table 4:** Strain in culprit versus remote segments.

**IRA (segments)**	**Strain direction %**	**Remote segments**	**Culprit segments**	***p***
**LAD (1, 2, 7, 8, 13, 14)**		***n* = 130**	***n* = 78**	
	**SCS**			
	Fast-SENC	−15 (7)	−8 (6)	<0.01
	FT	−13 (8)	−9 (9)	<0.01
	**SLS**			
	Fast-SENC	−17 (7)	−9 (6)	<0.01
	FT SLS	−14 (11)	−9 (6)	<0.01
**LCX (5, 6, 11, 12, 16)**		***n* = 66**	***n* = 30**	
	**SCS**			
	Fast-SENC	−16 (5)	−15 (6)	0.41
	FT	−16 (8)	−13 (7)	0.04
	**SLS**			
	Fast-SENC	−15 (6)	−15 (5)	0.23
	FT	−13 (8)	−16 (9)	0.37
**RCA (3, 4, 9, 10, 15)**		***n* = 121**	***n* = 55**	
	**SCS**			
	Fast-SENC	−18 (6)	−11 (6)	<0.01
	FT	−17 (7)	−11 (9)	<0.01
	**SLS**			
	Fast-SENC	−17 (5)	−14 (6)	0.01
	FT	−15 (7)	−13 (8)	<0.01

Segmental strains for infarcted related artery (IRA) and remote segments with means, standard deviation (SD) in parenthesis with p-values are presented. The 17-segment model of the American Heart Association, excluding the apical cap (segment 17) was used for left anterior descending artery (LAD), left circumflex artery (LCX) and right coronary artery (RCA).

**Table 5:** Correlations of segmental strain.

**IRA**	**Correlation variables**	**r (df)**	***p***
**LAD segments (*n* = 180)**			
**Segments: 1, 2, 7, 8, 13, 14**	**SCS vs. MI scar**		
	Fast-SENC	0.65 (178)	<0.01
	FT	0.59 (178)	<0.01
	**SLS vs. MI scar**		
	Fast-SENC	0.48 (178)	<0.01
	FT SLS	0.40 (178)	<0.01
	2DEcho	0.54 (178)	<0.01
**LCX segments (*n* = 150)**			
**Segments: 5, 6, 11, 12, 16**	**SCS vs. MI scar**		
	Fast-SENC	0.44 (148)	<0.01
	FT	0.24 (148)	<0.01
	**SLS vs. MI scar**		
	Fast-SENC	0.33 (148)	<0.01
	FT	0.20 (148)	0.01
	2DEcho	0.29 (148)	<0.01
**RCA segments (*n* = 150)**			
**Segments: 3, 4, 9, 10, 15**	**SCS vs. MI scar**		
	Fast-SENC	0.50 (148)	<0.01
	FT	0.25 (148)	<0.01
	**SLS vs. MI scar**		
	Fast-SENC	0.40 (148)	<0.01
	FT	0.25 (148)	<0.01
	2Decho	0.32 (148)	<0.01

Segmental strain for infarcted related artery (IRA) with Pearson correlation coefficient r (df = N-2) to myocardial infarction transmurality (MI scar) with p-values are presented. The 17-segment model of the American Heart Association was used, excluding the apical cap (segment 17), for each coronary artery.

### Receiver operating characteristics analysis

**Figure 8:**
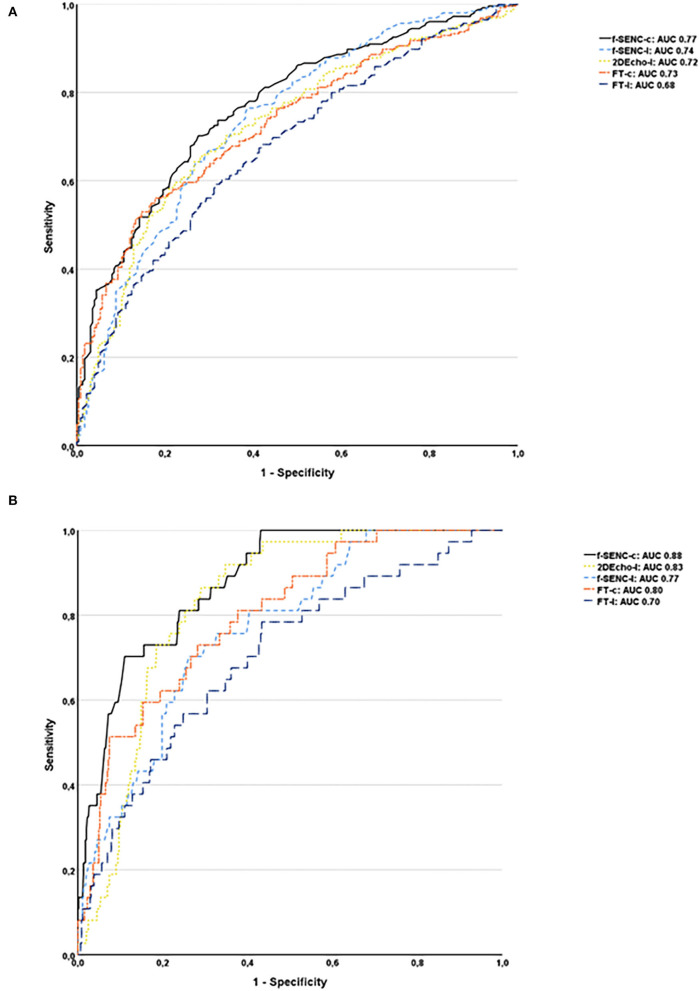
Receiver operator characteristics analysis of all infarcted segments **(A)** and segments with the transmurality >50% for fast-SENC, FT, and 2DEcho are presented with circumferential strain (-c), segmental longitudinal strain (-l), and area under the curve (AUC) **(B)**.

Fast-SENC had a higher AUC for detecting infarcted segments than FT for both SCS and SLS. SCS derived from fast-SENC detected segments with scar transmurality >50%, with the highest sensitivity (73%) at a specificity of 80% and AUC (0.88). SLS derived from 2DEcho detected scar transmurality >50%, with the sensitivity of 73% at specificity of 80%, and AUC of 0.83, Figure 8.

## Acquisition and reproducibility

All patients had good image quality for fast-SENC, cine bSSFP, LGE, and 2DEcho. The end-systolic phase was captured at 304 (SD 33 ms) ms after the R-wave for cine images, and at 303 (SD 34 ms) ms for the fast-SENC mages. The fast-SENC acquisition took 120 (SD 30 s) s and its post-processing 213 (SD 17 s) s, Table 6. The acquisition of the FT took 180 (60 s) s, and the post-processing took 150 (30 s). The fast-SENC interobserver reproducibility for GCS and GLS had an ICC of 0.98 (CI 0.97–0.99) and 1.00 (CI 0.99–1.00), and intraobserver reproducibility of ICC 0.98 (CI 0.95–0.99) and 1.00 (CI 0.99–1.00) for GCS and GLS respectively, Table 6. The interobserver reproducibility for FT GCS and GLS was ICC 0.96 (CI 0.89–0.99) and 0.98 (CI 0.94–1.0), Table 6.

**Table 6:** Time spent in data collection and post-processing.

	**Acquisition** **time (s)**	**Post processing** **time (s)**	**Interobserver** **reproducibility** **GCS GLS**
Fast-SENC	120 (30)	213 (17)	0.98 (CI 0.97–0.99)
			1.00 (CI 0.99–1.00)
FT	180 (60)	150 (30)	0.96 (CI 0.89–0.99)
			0.98 (CI 0.94–1.00)

Acquisition and post processing time with means and standard deviation (SD) in parenthesis. Interobserver reproducibility for global circumferential strain (GCS) and global longitudinal strain (GLS) with 95% confidence intervals are shown.

## Discussion

We performed a head-to-head comparison of myocardial strain assessment between fast-SENC and FT in post-STEMI patients. We were able to demonstrate good interobserver reproducibility and high correlations between the MR techniques with minor differences comparing GLS_CMR_ to GLS of 2DEcho. This is in line with Bucius et al. who found high global strain correlations between fast-SENC, FT and myocardial tagging but who also presented a considerably greater bias between the methods than shown in our study. Furthermore, Obokata et al. also demonstrated high correlation and fairly wide limits of agreement between FT and speckle tracking echocardiography (25, 26).

The assessment of LV contractile dysfunction after a STEMI has important prognostic relevance (11). Although LVEF is an important parameter post-MI, it may not be sufficiently sensitive for detecting subtle changes (6). The myocardial strain has been found to decline earlier than LVEF, which makes it an important complementing method for the evaluation of the LV (25). Many of the segments in our study had a scar transmurality <25%, so only subtle wall motion abnormalities should be expected. Still, we were able to detect significant differences in strain between IRA segments compared to the remote myocardium. This illustrates that strain measurement after myocardial infarction (MI) could possibly be useful for risk stratification of patients.

We were also able to demonstrate high correlations, with slightly higher absolute values for fast-SENC compared to FT and 2DEcho, in the detection of scar segments in the three perfusion territories. Few gadolinium-free alternatives exist for the detection of infarcted myocardial regions, but GCS has been proposed for this task (14). In our study, we could confirm this correlation between GCS and infarcted segments at a level similar to that in previous studies (14).

Both MR deformation methods provide a rapid and objective assessment of myocardial function, which makes them viable alternatives to other time-consuming MR procedures. Additional larger studies with patient follow-up could further identify conditions related to the development of adverse remodeling in subjects with reduced strain values.

## Conclusion

Fast-strain encoding showed higher sensitivity and specificity for detecting infarcted segments than FT. Segmental strain calculated for the perfusion territory of the infarct-related artery showed significantly lower strain values compared to the remote myocardium and this correlated with infarct transmurality. This study was not designed to explore the reproducibility of segmental strain values, but for global strain measurement, excellent reproducibility was detected. The GLS and GCS did not differ significantly between the two methods. Both MR methods showed acceptable clinical agreement with speckle tracking GLS obtained from echocardiography. The acquisition time of fast-SENC was very short, facilitating the investigation of patients with respiratory compromise.

## Limitations

This was a study of STEMI patients early after the pPCI and the results may not be applicable in all situations of reduced systolic LV function. Most infarcted segments had MI transmurality <25% which may result in a very subtle lowering of strain. The relatively low number of participants also limits the conclusions. The participating patients were somewhat younger and the male proportion was larger than average for STEMI patients in our catchment area. The presence of risk factors was typical, but the reporting of a family history of cardiac disease in first-degree relatives was probably underrepresented or forgotten by the patients. Adding tagging or displacement encoding with stimulated echoes would have complemented the assessment of deformation measurements using CMR. The present study was limited to the acute phase of STEMI treatment and did not include patient follow-up. We have used the standard AHA definition of perfusion territories, but variation between left- and right-dominated coronary vessel anatomies may especially affect the partition of segments between the LCX and RCA territories, which could have weakened the associations in our evaluation.

## Data availability statement

The datasets presented in this article are not readily available because of privacy concerns of the patients. However, data can be made available through the corresponding author, upon reasonable request. Requests to access the datasets should be directed to walid.el-saadi@liu.se.

## Ethics statement

The studies involving human participants were reviewed and approved by the Swedish Ethical Review Authority in Uppsala, registration number 2019-00480. The patients/participants provided their written informed consent to participate in this study.

## Author contributions

JE, JK, TE, MM, and SF participated in the design method and CMR acquisition, to ensure high-quality images. JE included patients, reviewed the manuscript, coordinated, and supported the study with research funding. WE-S, J-EK, and JK analyzed and interpreted the data, performed the statistical analysis, and wrote the manuscript with the assistance of all the other co-authors. JA and SS revised the manuscript for important related content and helped with the interpretation of the data and results. All authors made relevant contributions to the study. Before publication, the manuscript was reviewed and approved by all authors.

## Funding

Financial support for WE-S was obtained from Futurum—the unit for research and education, the academy for health and care in County Hospital Jönköping, and the Research Council of Southeastern Sweden. The study was supported by the Faculty of Medicine and Health Sciences, Linköping University, Sweden. This study was not supported by any specific funding that could have influenced the results.

## Conflict of interest

The authors declare that the research was conducted in the absence of any commercial or financial relationships that could be construed as a potential conflict of interest.

## Publisher's note

All claims expressed in this article are solely those of the authors and do not necessarily represent those of their affiliated organizations, or those of the publisher, the editors and the reviewers. Any product that may be evaluated in this article, or claim that may be made by its manufacturer, is not guaranteed or endorsed by the publisher.
