# Corrigendum: A head-to-head comparison of myocardial strain by fast-strain encoding and feature tracking imaging in acute myocardial infarction

**DOI:** 10.3389/fcvm.2023.1140214

**Published:** 2023-02-02

**Authors:** Walid El-Saadi, Jan Edvin Engvall, Joakim Alfredsson, Jan-Erik Karlsson, Marcelo Martins, Sofia Sederholm, Shaikh Faisal Zaman, Tino Ebbers, Johan Kihlberg

**Affiliations:** ^1^Department of Internal Medicine, Ryhov County Hospital, Region Jönköping County, Jönköping, Sweden; ^2^Department of Health, Medicine and Caring Sciences, Linköping University, Linköping, Sweden; ^3^Department of Clinical Physiology in Linköping and Department of Health, Medicine and Caring Sciences, Linköping University, Linköping, Sweden; ^4^Center for Medical Imaging Science and Visualization, Linköping University, Linköping, Sweden; ^5^Department of Cardiology in Linköping and Department of Health Medicine and Caring Sciences, Linköping University, Linköping, Sweden; ^6^Department of Radiology in Linköping and Department of Health Medicine and Caring Sciences, Linköping University, Linköping, Sweden

**Keywords:** cine magnetic resonance imaging, myocardial ischemia, ST elevation myocardial infarction, myocardial stunning, left ventricular dysfunction, left ventricular remodeling

In the published article, measurements in Table 2 were displaced. A formatting error resulted in strain percentage with confidence interval for the first and third lines were displayed upwards. The published Table 2 is printed below, followed by the corrected Table 2 and its caption.

Faulty Table 2:

In the published article, three figures that displayed SD in the first paragraph under “Results” were printed as hyperlinks to the list of references.

The authors wish that a correction is made to “Results, scar and ejection fraction” in terms of removing the hyperlinks displayed below. These sentences previously stated:

“**Scar and ejection fraction**

The subjects were enrolled and treated with the pPCI after identification of the culprit artery in each case. The cohort displayed a median door-to-balloon time of 67 min. The average scar size was 15 (9) % of LVM with a median Troponin-T of 1,640 ng/l, equivalent to 164 × upper level of normal. LGE revealed scars in 240 out of 510 segments (47%) with 122 segments having scar transmurality <25%, 78 segments between 25 and 49%, and only 40 segments had a transmurality ≥50%. In 13 patients the LVEF_CMR_ was little affected, LVEF_CMR_ ≥ 50%. Patients with maintained LVEF_CMR_ had smaller scar size 10 (5)% than those with depressed LVEF_CMR_ <50% whose scar size was 19 (10)%, (*p* < 0.01). Patient demographics and CMR imaging characteristics are presented in Table 1.”

The corrected sentences appears below:

“The subjects were enrolled and treated with pPCI after identification of the culprit artery in each case. The cohort displayed a median door-to-balloon time of 67 min. Average scar size was 15 (9) % of LVM with a median Troponin-T of 1,640 ng/l, equivalent to 164 x upper level of normal. LGE revealed scar in 240 out of 510 segments (47%) with 122 segments having scar transmurality < 25%, 78 segments between 25 and 49% and only 40 segments had a transmurality ≥ 50%. In 13 patients the LVEF_CMR_ was little affected, LVEF_CMR_ ≥ 50%. Patients with maintained LVEF_CMR_ had smaller scar size 10 (5) % than those with depressed LVEF_CMR_ < 50% whose scar size was 19 (10) %, (*p* < 0.01). Patient demographics and CMR imaging characteristics are presented in Table 1.”

The authors apologize for this error and state that this does not change the scientific conclusions of the article in any way. The original article has been updated.

**Faulty** Table 2:

**Table 2:** Global circumferential and longitudinal strain.

**Myocardial strain direction**	**Mean % (*SD*)**	**95% CI for mean**
**GCS**	−13.6 (3.7)	−14.9 to −12.2
Fast-SENC	
FT	−13.6 (3.7)	−15.0 to −12.2
**GLS**	−14.8 (2.9)	−15.9 to −13.7
Fast-SENC	
FT	−13.0 (2.8)	−14.0 to −11.9
2DEcho	−13.3 (3.7)	−14.7 to −11.9

Global circumferential strain (GCS) and global longitudinal strain (GLS) derived from fast-SENC, FT and 2DEcho. Means and standard deviation (SD) in parenthesis with 95% confidence intervals are presented.

**Corrected** Table 2:

**Table 2:** Global circumferential and longitudinal strain.

**Myocardial strain direction**	**Mean % (SD)**	**95% CI for mean**
**GCS**		
Fast-SENC	−13.6 (3.7)	−14.9 to −12.2
FT	−13.6 (3.7)	−15.0 to −12.2
**GLS**		
Fast-SENC	−14.8 (2.9)	−15.9 to −13.7
FT	−13.0 (2.8)	−14.0 to −11.9
2DEcho	−13.3 (3.7)	−14.7 to −11.9

Global circumferential strain (GCS) and global longitudinal strain (GLS) derived from fast-SENC, FT and 2DEcho. Means and standard deviation (SD) in parenthesis with 95% confidence intervals are presented.

